# Coil-Assisted Onyx Embolization of an Ophthalmic Artery-Supplied Anterior Cranial Fossa Dural Arteriovenous Fistula Mimicking an Intracranial Aneurysm

**DOI:** 10.7759/cureus.109949

**Published:** 2026-05-30

**Authors:** Yogesh Shahi, Adarsha Mahaseth, Lv Penghua, Ma Yuan, Yan Lele, Wang Fu-an

**Affiliations:** 1 Department of Interventional Radiology, Northern Jiangsu People's Hospital Affiliated to Yangzhou University, Yangzhou, CHN; 2 Department of Internal Medicine, Nepalese Army Institute of Health Sciences, Kathmandu, NPL

**Keywords:** anterior cranial fossa, anterior ethmoidal artery, coil-assisted embolization, cortical venous drainage, digital subtraction angiography, dural arteriovenous fistula, intracranial aneurysm mimic, onyx embolization, ophthalmic artery, venous varix

## Abstract

Dural arteriovenous fistulas are acquired intracranial vascular malformations characterized by abnormal shunting between dural arterial feeders and venous structures. Anterior cranial fossa and ethmoidal dural arteriovenous fistulas are uncommon but clinically important lesions because they frequently drain directly into cortical veins and may be associated with venous ectasia or aneurysmal venous dilatation. When venous variceal dilatation is present, these lesions may mimic intracranial aneurysms on noninvasive vascular imaging, making digital subtraction angiography essential for definitive diagnosis and treatment planning.

We report the case of an 84-year-old man who presented with slurred speech and left-sided limb weakness. Magnetic resonance imaging demonstrated bilateral chronic ischemic white matter changes and lacunar infarct-related signal abnormalities; therefore, the acute presentation was initially attributed to ischemic cerebrovascular disease. However, computed tomography angiography more clearly demonstrated abnormal vessels with localized aneurysm-like dilatation in the right frontal/anterior cranial fossa region, prompting further angiographic evaluation. Digital subtraction angiography revealed an anterior cranial fossa dural arteriovenous fistula supplied by bilateral ophthalmic arteries, predominantly from the right side, with direct cortical venous drainage and aneurysmal venous dilatation in the right frontal region. These findings classified the lesion as a high-grade dural arteriovenous fistula corresponding to Borden type III and Cognard type IV.

The patient underwent endovascular treatment under general anesthesia. The dominant right ophthalmic/anterior ethmoidal arterial feeder was selectively catheterized using an Echelon microcatheter over a Synchro microguidewire. Axium Prime detachable coils were deployed in the mid-to-distal arterial segment distal to the origin of the central retinal artery, followed by a controlled injection of Onyx-18. Final angiography demonstrated no residual arteriovenous shunting, complete disconnection of the aneurysmally dilated venous pouch from the arterial feeder, and no early venous filling during the arterial phase. The postoperative course was uneventful, with no clinically evident new neurological or visual complications.

This case highlights the diagnostic importance of digital subtraction angiography in aneurysm-mimicking anterior cranial fossa vascular lesions and demonstrates that coil-assisted Onyx embolization may be feasible in carefully selected ophthalmic/anterior ethmoidal artery-supplied dural arteriovenous fistulas when safe distal catheterization and controlled embolic delivery can be achieved.

## Introduction

Dural arteriovenous fistulas are acquired intracranial vascular malformations characterized by an abnormal shunting between dural arterial feeders and venous drainage pathways. Unlike brain arteriovenous malformations, they lack a compact intraparenchymal nidus. Their clinical behavior is mainly determined by the venous drainage pattern, particularly the presence of cortical venous reflux, direct cortical venous drainage, venous ectasia, or variceal dilatation [1-5].

Anterior cranial fossa and ethmoidal dural arteriovenous fistulas are rare but high-risk lesions. They are commonly formed in the anterior or posterior ethmoidal branches of the ophthalmic artery and often drain directly into cortical veins rather than a dural venous sinus [6]. When these fistulas are associated with venous varix or aneurysmal venous dilatation, the lesions may mimic intracranial aneurysms on computed tomography angiography or magnetic resonance angiography [7,8]. Digital subtraction angiography is therefore essential for defining the arterial supply, fistulous point, venous drainage pattern, and treatment strategy [2,6].

Although microsurgical disconnection has traditionally been considered a definitive treatment, advances in microcatheter technology, liquid embolic agents, and distal ophthalmic artery navigation have expanded the role of endovascular therapy in selected cases [9-13]. However, embolization through the ophthalmic artery territory remains technically demanding because reflux or unintended migration of embolic material may compromise retinal or optic nerve circulation [10,13,14].

We report the case of an elderly patient with an ophthalmic/anterior ethmoidal artery-supplied anterior cranial fossa dural arteriovenous fistula that mimicked an intracranial aneurysm on computed tomography angiography. The patient was treated successfully with coil-assisted Onyx embolization.

## Case presentation

An 84-year-old man presented with a sudden onset of slurred speech and left-sided limb weakness for five days. But there was no associated headache, visual disturbance, seizure-like activity, or altered level of consciousness. He had a history of hypertension for three years, which was controlled with benidipine hydrochloride, and a previous history of surgery for intestinal obstruction. There was no known history of diabetes mellitus, coronary artery disease, or other major systemic illness.

Before presentation to our center, cranial magnetic resonance imaging performed at another hospital demonstrated bilateral chronic ischemic white matter changes and lacunar infarct-related signal abnormalities (Figure 1). The patient’s acute neurological symptoms were therefore initially considered more likely related to ischemic cerebrovascular disease rather than directly attributable to a vascular malformation. On a retrospective review, the aneurysm-like venous dilatation may have been subtly observable on the initial magnetic resonance imaging; however, subsequent head and neck computed tomography angiography more clearly highlighted abnormal vessels with localized aneurysm-like vascular dilatation in the right frontal/anterior cranial fossa region. This finding prompted further angiographic evaluation to distinguish a true intracranial aneurysm from another vascular lesion (Figure 2).

**Figure 1:**
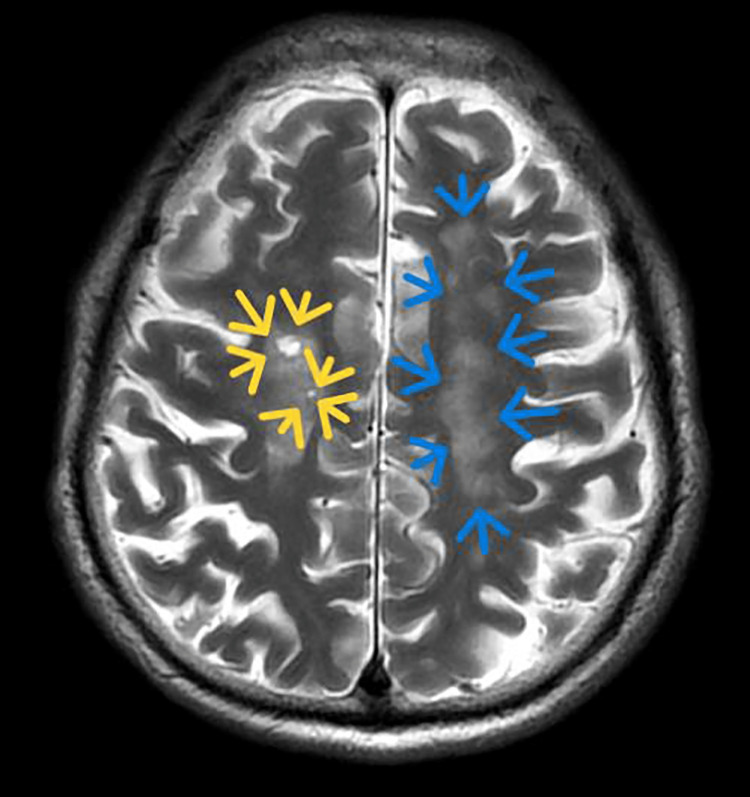
Pre-treatment brain magnetic resonance imaging. Axial T2-weighted magnetic resonance image demonstrating bilateral chronic ischemic white matter changes and lacunar infarct-related signal abnormalities. Yellow arrows indicate focal lacunar/ischemic lesions, while blue arrows indicate more diffuse periventricular and subcortical white matter hyperintensities.

**Figure 2:**
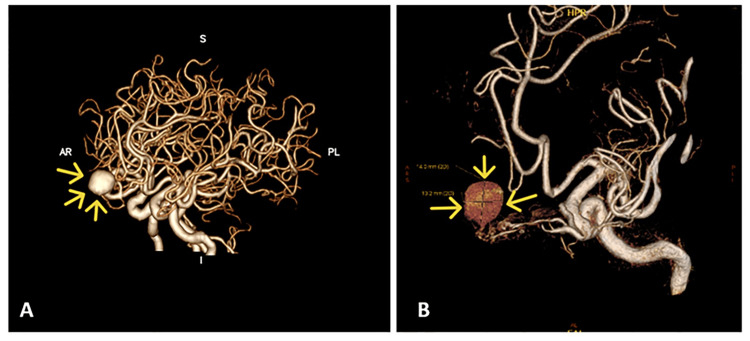
Pre-embolization computed tomography angiography showing an aneurysm-like vascular abnormality. (A) Three-dimensional reconstructed computed tomography angiography demonstrating abnormal vascular dilatation in the right frontal region, initially raising suspicion for a localized intracranial aneurysm (arrow). (B) Measurement image showing an elongated aneurysm-like vascular dilatation measuring approximately 40.0×13.2 mm (arrow), which was subsequently confirmed on digital subtraction angiography to represent aneurysmal venous dilatation associated with an anterior cranial fossa dural arteriovenous fistula rather than a true arterial aneurysm.

On admission, the patient was conscious and clinically stable. Neurological examination showed mild left-sided weakness, with Medical Research Council grade 4+ power in the left-sided limbs and grade 5 power in the right-sided limbs. Pupils were equal and reactive to light, and there were no meningeal signs or clinically evident visual symptoms. Based on the clinical presentation and initial imaging findings, the working diagnoses were cerebral infarction, suspected intracranial aneurysm, and hypertension.

Baseline laboratory investigations are summarized in Table 1. There was no major hematologic, coagulation, hepatic, renal, or electrolyte abnormality that contraindicated angiographic evaluation or endovascular treatment.

**Table 1:** Baseline laboratory investigations. RBC: Red blood cell count; CV: coefficient of variation; SD: standard deviation; HBsAg: hepatitis B surface antigen; HBeAg: hepatitis B e antigen; HIV: human immunodeficiency virus; HCV: hepatitis C virus; TPAB: Treponema pallidum antibody.

Parameter	Result	Reference range
Red blood cell count	3.88×10¹²/L	4.3-5.8×10¹²/L
Hemoglobin	126 g/L	130-175 g/L
Hematocrit	37%	40-50%
Mean corpuscular volume	95.4 fL	82-100 fL
Mean corpuscular hemoglobin	32.4 pg	27-34 pg
Mean corpuscular hemoglobin concentration	341 g/L	316-354 g/L
Red cell distribution width - CV	14.3%	11.7-15.3%
Red cell distribution width - SD	50.5 fL	38.4-47.8 fL
White blood cell count	4.95×10⁹/L	3.5-9.5×10⁹/L
Neutrophils	72.7%	40-75%
Lymphocytes	15.6%	20-50%
Monocytes	8.9%	3-10%
Eosinophils	2.5%	0.4-8%
Basophils	0.3%	0-1%
Absolute neutrophil count	3.60×10⁹/L	1.8-6.3×10⁹/L
Absolute lymphocyte count	0.77×10⁹/L	1.1-3.2×10⁹/L
Platelet count	157×10⁹/L	125-350 × 10⁹/L
Mean platelet volume	9.3 fL	8.04-12.7 fL
Plateletcrit	0.146%	0.169-0.374%
Prothrombin time	11.9 s	9-13 s
International normalized ratio	1.04	0.75-1.25
Activated partial thromboplastin time	27.3 s	23.3-32.5 s
Thrombin time	16.7 s	14-21 s
Fibrinogen	2.25 g/L	2-4 g/L
Albumin	36.5 g/L	35-50 g/L
Globulin	32.1 g/L	20-30 g/L
Albumin/globulin ratio	1.1	1.1-2.5
Total bilirubin	12.3 µmol/L	3-22 µmol/L
Indirect bilirubin	7.6 µmol/L	0-19 µmol/L
Direct bilirubin	0.0 µmol/L	0-5 µmol/L
Alanine aminotransferase	10 U/L	<50 U/L
Aspartate aminotransferase	20 U/L	17-59 U/L
Urea nitrogen	4.89 mmol/L	1.7-8.3 mmol/L
Creatinine	78 µmol/L	58-110 µmol/L
Uric acid	199 µmol/L	90-450 µmol/L
Total carbon dioxide	22.50 mmol/L	18-30 mmol/L
Potassium	4.03 mmol/L	3.5-5.1 mmol/L
Sodium	139 mmol/L	137-145 mmol/L
Chloride	106.8 mmol/L	98-107 mmol/L
Calcium	2.12 mmol/L	2.1-2.55 mmol/L
Estimated glomerular filtration rate	78.97 mL/min/1.73 m²	>90 mL/min/1.73 m²
HBsAg, HBeAg, HIV, HCV, and TPAB	Negative	Negative
Hepatitis B surface antibody	2.640, negative	0-10 IU/L
Hepatitis B e antibody	1.5, negative	Negative

Diagnostic cerebral angiography was performed to clarify the lesion morphology and guide management. The procedure was performed using the modified Seldinger technique by employing a right femoral arterial access. Aortic arch angiography revealed the origins of the brachiocephalic trunk, left common carotid artery, and left subclavian artery. Selective angiography of the bilateral carotid and vertebrobasilar circulations was then performed.

Cerebral angiography showed an enlargement of the bilateral ophthalmic arteries, disorganized distal vascular channels, direct cortical venous drainage, and early opacification of local draining veins during the arterial phase. Aneurysmal dilatation of the draining venous structure was identified, more prominently on the right side, with the venous aneurysmal component located in the right frontal region. These findings pointed to an anterior cranial fossa dural arteriovenous fistula supplied by bilateral ophthalmic/anterior ethmoidal arterial branches, with right-sided predominance (Figure 3). Based on direct cortical venous drainage and aneurysmal venous dilatation, the lesion was classified as a high-grade dural arteriovenous fistula corresponding to Borden type III and Cognard type IV. The initial suspicion of an intracranial aneurysm was therefore revised to an anterior cranial fossa dural arteriovenous fistula with aneurysmal venous dilatation.

**Figure 3:**
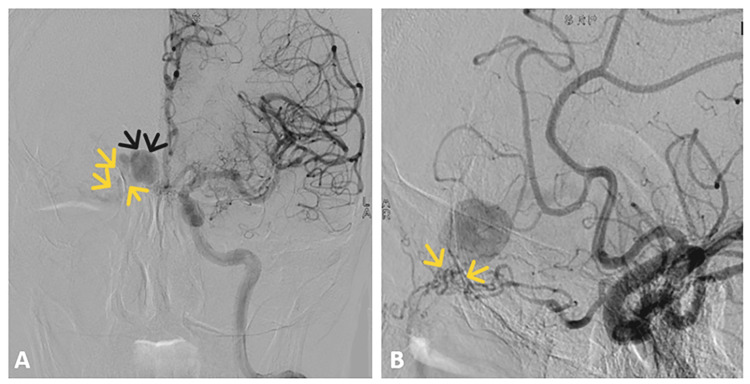
Diagnostic cerebral angiography confirming a high-grade anterior cranial fossa dural arteriovenous fistula. (A) Selective cerebral angiography demonstrating hypertrophied ophthalmic/anterior ethmoidal arterial supply to the anterior cranial fossa dural arteriovenous fistula, with aneurysmal dilatation of the draining venous structure in the right frontal region (arrows). (B) Arterial-phase angiographic image showing early cortical venous opacification through the fistulous connection, confirming direct cortical venous drainage; together with aneurysmal venous dilatation, these findings classify the lesion as a high-grade dural arteriovenous fistula corresponding to Borden type III and Cognard type IV (arrows).

Following confirmation of the diagnosis, treatment options were discussed with the patient’s family. Both endovascular embolization and microsurgical disconnection were explained, including their potential benefits and risks. Because of the patient’s advanced age, the presence of a dominant technically accessible right-sided ophthalmic/anterior ethmoidal feeder, and the family’s preference for a less invasive approach, endovascular embolization was selected.

The embolization procedure was performed under general anesthesia. Right femoral arterial access was obtained, and an 8F sheath was placed. A guiding catheter was advanced into the right internal carotid artery. Repeat angiography and three-dimensional imaging demonstrated enlargement of the right ophthalmic artery, early cortical venous drainage, and aneurysmal venous dilatation in the right frontal region. Using an Echelon microcatheter (Medtronic Neurovascular, Irvine, CA, USA) over a Synchro microguidewire (Stryker Neurovascular, Fremont, CA, USA), the dominant right ophthalmic/anterior ethmoidal arterial feeder was selectively catheterized. The microcatheter was advanced into a stable mid-to-distal position beyond the origin of the central retinal artery, allowing embolization to be performed distal to the major retinal arterial supply (Figure 4A).

After stable distal catheter positioning was achieved, two Axium Prime detachable coils (Medtronic Neurovascular, Irvine, CA, USA) measuring 2 mm×60 mm and 1.5 mm×20 mm were deployed in the mid-to-distal segment of the ophthalmic/anterior ethmoidal arterial feeder, distal to the origin of the central retinal artery. Coil deployment was used as an adjunct to control embolic flow, reduce the risk of uncontrolled reflux or migration, and facilitate targeted embolization of the fistulous communication and associated venous pouch. Following microcatheter preparation with dimethyl sulfoxide, a controlled injection of Onyx-18 liquid embolic system (Medtronic Neurovascular, Irvine, CA, USA) was undertaken. A total of 1.6 mL of Onyx-18 was injected to occlude the fistulous site and disconnect the aneurysmally dilated venous pouch from the arterial feeder (Figure 4B). A total of 5,000 units of heparin was administered during the procedure.

**Figure 4:**
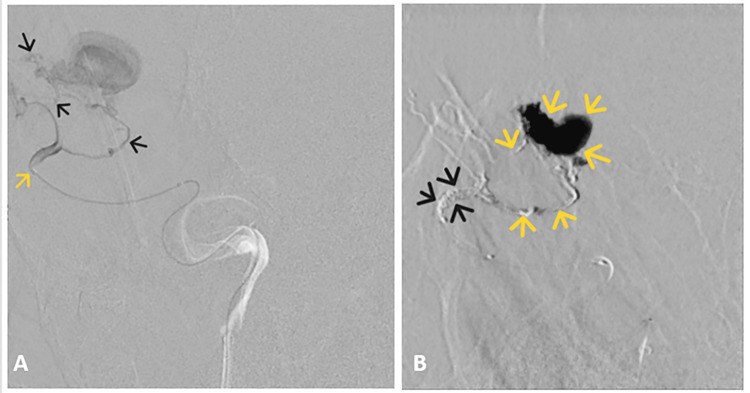
Intraoperative angiographic images during coil-assisted Onyx-18 embolization. (A) Selective angiography after microcatheter navigation demonstrating positioning of the Echelon microcatheter within the dominant right ophthalmic/anterior ethmoidal arterial feeder, with the catheter tip located in the mid-to-distal arterial segment beyond the origin of the central retinal artery (arrows). (B) Angiographic image obtained after coil deployment and controlled injection of 1.6 mL of Onyx-18 showing embolization of the fistulous communication and associated venous pouch/aneurysmal venous dilatation; the coils were deployed distal to the origin of the central retinal artery to assist flow control and facilitate targeted Onyx embolization (arrows).

Final post-embolization digital subtraction angiography showed a complete immediate angiographic occlusion of the lesion. There was no residual arteriovenous shunting, complete disconnection of the venous pouch from the arterial feeder, and no early venous filling during the arterial phase. Distal arterial flow was preserved, and flow through the right internal carotid and middle cerebral arteries remained smooth. Subsequent left internal carotid artery angiography showed no persistent contralateral fistulous filling, confirming angiographic elimination of the previously demonstrated bilateral ophthalmic arterial contribution (Figure 5). The procedure was completed successfully, and the puncture site was closed using a Perclose ProGlide closure device (Abbott Vascular, Santa Clara, CA, USA).

**Figure 5:**
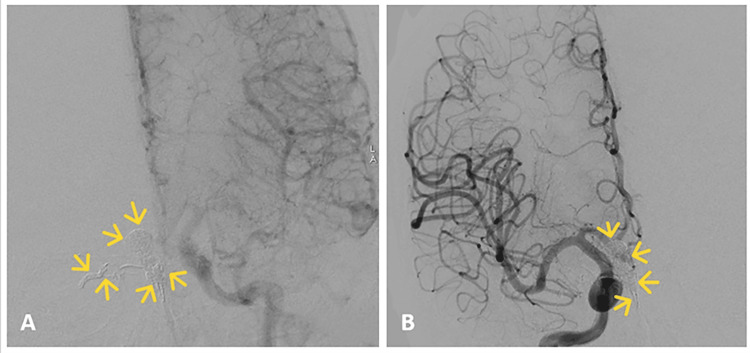
Final post-embolization cerebral angiography demonstrating complete immediate angiographic occlusion. (A) Right internal carotid artery angiography after coil-assisted Onyx-18 embolization demonstrating no residual arteriovenous shunting, complete disconnection of the aneurysmally dilated venous pouch from the arterial feeder, absence of early venous filling during the arterial phase, and preserved distal flow through the right internal carotid and middle cerebral arteries. (B) Left internal carotid artery angiography showing no persistent contralateral ophthalmic arterial supply, cross-filling, or residual early venous drainage, confirming complete immediate angiographic obliteration of the previously bilateral ophthalmic artery-supplied fistula.

The postoperative course was uneventful. The patient remained hemodynamically stable, with no new neurological deficit or clinically evident visual complication. He did not report blurring of vision, diplopia, ocular pain, or visual field symptoms. Bedside examination showed equal and reactive pupils, full extraocular movements, and clinically preserved vision and visual fields bilaterally. Because there were no subjective visual complaints or clinical evidence of visual impairment, formal ophthalmological evaluation was not performed during the index admission.

The patient was discharged on the fourth postoperative day in a stable condition. He was advised to return for clinical review and follow-up cerebral angiography after three months to assess the durability of fistula occlusion and exclude residual or recurrent arteriovenous shunting.

## Discussion

This case illustrates the diagnostic and therapeutic challenges associated with anterior cranial fossa and ethmoidal dural arteriovenous fistulas. The patient initially presented with acute neurological symptoms suggestive of ischemic cerebrovascular disease, and cranial magnetic resonance imaging showed multiple lacunar infarct-related signal abnormalities and chronic ischemic white matter changes. Therefore, the patient’s slurred speech and left-sided weakness were considered more likely related to ischemic cerebrovascular disease rather than directly attributable to the dural arteriovenous fistula. On a retrospective review, the aneurysm-like venous dilatation may have been subtly observable on the initial magnetic resonance imaging; however, computed tomography angiography more clearly demonstrated an aneurysm-like vascular abnormality in the right frontal/anterior cranial fossa region. Digital subtraction angiography revealed that this lesion was not a conventional arterial aneurysm but an anterior cranial fossa dural arteriovenous fistula supplied by bilateral ophthalmic/anterior ethmoidal arterial branches, predominantly on the right side, with direct cortical venous drainage and aneurysmal venous dilatation.

The aneurysm-mimicking appearance in this case is consistent with previous reports. Chen et al. described an anterior cranial fossa dural arteriovenous fistula that angiographically mimicked an anterior ethmoidal artery aneurysm, with operative confirmation that the suspected aneurysmal lesion was a thrombosed venous varix [7]. Ogawa et al. similarly reported an anterior cranial fossa dural arteriovenous fistula with a varix mimicking an anterior communicating artery aneurysm on computed tomography and magnetic resonance imaging [8]. These reports, together with the present case, emphasize that aneurysm-like vascular lesions in the anterior cranial fossa should be evaluated carefully with digital subtraction angiography before definitive treatment is planned.

The angioarchitecture in this case was characteristic of an anterior cranial fossa/ethmoidal dural arteriovenous fistula. These lesions are commonly supplied by the anterior or posterior ethmoidal branches of the ophthalmic artery and often drain into cortical veins rather than a dural venous sinus [6]. This venous drainage pattern is clinically important because direct cortical venous drainage, venous ectasia, and venous variceal dilatation are associated with aggressive clinical behavior and increased hemorrhagic risk [2-5]. In the present case, the presence of direct cortical venous drainage and aneurysmal venous dilatation established the lesion as a high-grade dural arteriovenous fistula corresponding to Borden type III and Cognard type IV [3,4]. Although the patient did not present with documented intracranial hemorrhage, these angiographic features supported active treatment rather than conservative observation.

The mode of treatment for anterior cranial fossa/ethmoidal dural arteriovenous fistulas remains individualized. Microsurgical disconnection has traditionally been favored because these lesions are often located superficially along the anterior cranial base and can be definitively treated by interrupting the draining cortical vein at the fistulous site. Recent comparative studies continue to support microsurgery as a highly effective strategy. Berke et al., in a systematic review and meta-analysis of ethmoidal dural arteriovenous fistulas, found higher complete obliteration rates with microsurgery than with endovascular treatment and noted that some endovascularly treated patients required subsequent surgery [15]. Schimmel et al. similarly reported higher complete obliteration after microsurgery than endovascular embolization in anterior ethmoidal dural arteriovenous fistulas, supporting open surgery as a potentially more definitive option when complete fistula obliteration is the primary endpoint [16].

However, the role of endovascular therapy has expanded substantially. Xu et al. reported complete angiographic cure in 91.7% of endovascularly treated anterior cranial fossa dural arteriovenous fistulas, with good outcomes in 93.8% of patients [9]. Mayercik et al. evaluated embolization of dural arteriovenous fistulas through the ophthalmic artery and reported angiographic cure in 11 of 12 patients, with no permanent vision loss, death, or permanent disability [10]. Piergallini et al. reported complete obliteration and good outcomes in six consecutive patients with anterior cranial fossa dural arteriovenous fistulas treated using Onyx through the ophthalmic artery [11]. Ha et al. also reported successful Onyx embolization in ethmoidal dural arteriovenous fistulas supplied by ophthalmic artery branches [12]. More recent series by Puylaert et al. and Zhang et al. further support transarterial embolization as a feasible first-line approach in selected anterior cranial fossa dural arteriovenous fistulas [17,18]. These studies support endovascular therapy as a reasonable option when a safe distal microcatheter position can be achieved, reflux can be controlled, and embolic penetration into the fistulous point and proximal venous recipient can be obtained.

Bilateral ophthalmic arterial supply is relatively uncommon but has been described in high-risk anterior cranial fossa dural arteriovenous fistulas. Sirakov et al. reported a ruptured bilateral ophthalmic-frontal dural arteriovenous fistula presenting with subdural hematoma and severe epistaxis that was successfully treated endovascularly [19]. The present case adds to this literature by demonstrating bilateral ophthalmic arterial contribution with right-sided predominance and aneurysmal venous dilatation, but without overt hemorrhagic presentation. Although bilateral supply was demonstrated diagnostically, the right ophthalmic/anterior ethmoidal feeder represented the dominant and technically accessible arterial route. After coil-assisted Onyx embolization through the right-sided pedicle, repeat bilateral internal carotid artery angiography showed no residual arteriovenous shunting or early venous filling, including from the contralateral ophthalmic supply.

A key technical feature in the present case was the use of coil-assisted Onyx embolization through the dominant right ophthalmic/anterior ethmoidal arterial feeder. After selective catheterization with an Echelon microcatheter over a Synchro microguidewire, the microcatheter was positioned in the mid-to-distal arterial segment beyond the origin of the central retinal artery. Coils were then deployed distal to the central retinal artery origin to provide local flow control, reduce the risk of uncontrolled reflux or migration, and facilitate targeted embolization. Controlled Onyx injection was performed to occlude the fistulous communication and disconnect the aneurysmally dilated venous pouch from the arterial feeder. The final angiographic endpoint was achieved, with no residual arteriovenous shunting, complete disconnection of the venous pouch from the arterial feeder, and absence of early venous filling on arterial-phase digital subtraction angiography. Sugihara et al. also described a coil-assisted liquid embolic strategy in anterior cranial fossa dural arteriovenous fistula treatment, using coils to limit excessive liquid embolic migration and protect venous drainage, supporting the broader technical principle that coil assistance may improve control of embolic delivery in selected high-risk lesions [20].

Visual safety is a major consideration during embolization through the ophthalmic artery territory. Although the target feeder in this case was the anterior ethmoidal branch, reflux or non-target migration of embolic material within the ophthalmic arterial system may compromise ocular circulation. The ophthalmic artery supplies the retina and optic nerve through branches including the central retinal artery and posterior ciliary arteries, and embolic compromise of these branches may result in visual loss [10,13,14]. Kular et al. reported five anterior cranial fossa dural arteriovenous fistulas treated by micro-balloon-assisted trans-ophthalmic embolization, achieving complete immediate angiographic occlusion without visual field deficits and emphasizing protection of retinal and posterior ciliary circulation during liquid embolic delivery [13]. Conversely, Han et al. described posterior short ciliary artery ischemia after Onyx-18 embolization of an anterior ethmoidal artery-supplied dural arteriovenous fistula, underscoring the potential visual complications of this route despite technical angiographic success [14].

In the present case, embolization was performed from a mid-to-distal catheter position beyond the origin of the central retinal artery. Coil deployment and controlled Onyx injection were used to promote targeted embolization and minimize uncontrolled reflux. No clinically evident postoperative visual complication was observed. The patient did not report blurring of vision, diplopia, ocular pain, visual field defect, or other new visual symptoms. Bedside examination showed equal and reactive pupils, full extraocular movements, absence of nystagmus, and clinically preserved vision and visual fields bilaterally. However, subtle retinal or optic nerve ischemic changes cannot be completely excluded because formal preoperative and postoperative ophthalmological assessment, including visual acuity testing, fundus examination, and formal visual field testing, was not performed.

The patient’s advanced age was also relevant to treatment planning. Microsurgical disconnection may provide a definitive cure but requires craniotomy and carries perioperative risks, particularly in elderly patients. Endovascular treatment offered a less invasive option in this case, and the patient recovered without immediate neurological or clinically evident visual complication. However, this should not be interpreted to mean that all ophthalmic artery-supplied anterior cranial fossa dural arteriovenous fistulas are suitable for embolization. Treatment must be individualized according to fistula anatomy, arterial access, venous drainage pattern, relationship of the microcatheter position to ocular arterial branches, operator experience, and feasibility of controlled embolic delivery.

This report has several limitations. First, this is a single case and therefore cannot establish the comparative superiority of coil-assisted Onyx embolization over microsurgical disconnection, Onyx alone, n-butyl cyanoacrylate embolization, balloon-assisted embolization, or combined transarterial/transvenous approaches. Second, although immediate post-embolization digital subtraction angiography demonstrated complete occlusion with no residual arteriovenous shunting, complete disconnection of the venous pouch from the arterial feeder, and no early venous filling during the arterial phase, delayed angiographic follow-up was not yet available at the time of manuscript preparation. Therefore, the present report demonstrates complete immediate angiographic occlusion rather than proven long-term durable cure. Third, a formal ophthalmological examination was not performed because the patient had no visual complaints and bedside postoperative assessment showed clinically preserved vision and visual fields bilaterally. As a result, subtle retinal or optic nerve ischemic changes cannot be fully excluded.

## Conclusions

Anterior cranial fossa and ethmoidal dural arteriovenous fistulas are rare but potentially aggressive vascular lesions because they frequently drain directly into cortical veins and may be associated with venous ectasia or aneurysmal venous dilatation. These lesions may mimic intracranial aneurysms on computed tomography angiography or magnetic resonance angiography, particularly when a venous varix is present. Digital subtraction angiography remains essential for accurate diagnosis, angiographic classification, and treatment planning.

This case demonstrates complete immediate angiographic occlusion of an ophthalmic/anterior ethmoidal artery-supplied anterior cranial fossa dural arteriovenous fistula using coil-assisted Onyx embolization in an elderly patient. The lesion showed direct cortical venous drainage and aneurysmal venous dilatation, corresponding to a high-grade dural arteriovenous fistula classified as Borden type III and Cognard type IV. Coil deployment in the mid-to-distal ophthalmic/anterior ethmoidal arterial segment distal to the origin of the central retinal artery, followed by controlled Onyx injection, achieved complete immediate disconnection of the venous pouch from the arterial feeder. Although microsurgical disconnection remains an important definitive treatment option, modern endovascular therapy may be feasible in carefully selected patients when safe distal catheterization and controlled embolic delivery can be achieved. Follow-up cerebral angiography remains important to confirm durable occlusion and exclude residual or recurrent arteriovenous shunting.
